# A review of recent studies on the pathogenesis of Systemic Sclerosis: focus on fibrosis pathways

**DOI:** 10.3389/fimmu.2025.1551911

**Published:** 2025-04-16

**Authors:** Sergio A. Jimenez, Fabian A. Mendoza, Sonsoles Piera-Velazquez

**Affiliations:** ^1^ Jefferson Institute of Molecular Medicine and Scleroderma Center, Thomas Jefferson University, Philadelphia, PA, United States; ^2^ Division of Rheumatology, Department of Medicine, Thomas Jefferson University, Philadelphia, PA, United States

**Keywords:** Systemic Sclerosis, pathogenesis, myofibroblast, endothelial cell, miRNA - microRNA, lncRNA - long non-coding RNA, fibrosis, TGF

## Abstract

Systemic Sclerosis (SSc) is a systemic autoimmune disease of unknown etiology characterized by the development of frequently progressive cutaneous and internal organ fibrosis accompanied by severe vascular alterations. The pathogenesis of SSc is highly complex and, despite extensive investigation, has not been fully elucidated. Numerous studies have suggested that unknown etiologic factors cause multiple alterations in genetically receptive hosts, leading to SSc development and progression. These events may be functionally and pathologically interconnected and include: 1) Structural and functional microvascular and endothelial cell abnormalities; 2) Severe oxidative stress and high reactive oxygen species (3); Frequently progressive cutaneous and visceral fibrosis; 4) Transdifferentiation of various cell types into activated myofibroblasts, the cells ultimately responsible for the fibrotic process; 5) Establishment of a chronic inflammatory process in various affected tissues; 6) Release of cytokines, chemokines, and growth factors from the inflammatory cells; 7) Abnormalities in humoral and cellular immunity with the production of specific autoantibodies; and 8) Epigenetic alterations including changes in multiple non-coding RNAs. These events manifest with different levels of intensity in the affected organs and display remarkable individual variability, resulting in a wide heterogeneity in the extent and severity of clinical manifestations. Here, we will review some of the recent studies related to SSc pathogenesis.

## Highlights

- Systemic Sclerosis (SSc) is a systemic autoimmune disease characterized by a severe fibrotic process affecting the skin and multiple internal organs associated with a generalized obstructive vasculopathy of small arteries and arterioles.
- Despite extensive genetic, biochemical, and molecular studies, the exact pathogenetic mechanisms of SSc have not been fully elucidated, although several affected molecular pathways have been identified.
- The discovery of these molecular pathway alterations has led to a marked improvement in the understanding of SSc pathogenesis.
- Although there is no curative therapy for the disease, and it may progress, causing severe disabilities and high mortality, the recent identification of novel molecular therapeutic targets should be of value to optimize the treatment and reduce the mortality caused by the disease.

## Introduction

Systemic Sclerosis (SSc) is a clinically heterogeneous systemic autoimmune disease of unknown etiology characterized by a frequently progressive fibrotic process affecting the skin and various internal organs. The fibrotic process in SSc is usually accompanied by vasculopathy of small arteries and arterioles, the presence of a chronic inflammatory process in the affected tissues, and the occurrence of humoral and cellular immune abnormalities resulting in the production of multiple autoantibodies, some with high specificity for the disease and the SSc clinical phenotype (1–4).

The molecular mechanisms involved in the clinical and pathologic manifestations of the disease are highly complex, and although numerous studies have provided substantial information about its intricate picture and clarified some of its early events, the precise altered regulatory pathways involved have not been completely elucidated. However, it has been well recognized that SSc involves multiple alterations in various molecular pathways (5–9) that may occur simultaneously or may develop sequentially. These events include: 1) Fibroproliferative lesions of small arteries and arterioles accompanied by severe structural and functional endothelial cell alterations; 2) Severe oxidative and high reactive oxygen species; 3) Excessive and often progressive deposition of collagen and other extracellular matrix (ECM) macromolecules in skin and various internal organs; 4) Alterations of cellular and humoral immunity with the production of numerous autoantibodies, some with high disease and clinical phenotype specificity; 5) Establishment of a chronic inflammatory process in affected tissues; 6) Cellular transdifferentation resulting in the phenotypic conversion of various cell types including resting fibroblasts, endothelial cells, epithelial cells, adipocytes, and other cells into activated myofibroblasts, the cellular elements ultimately involved in the exaggerated and excessive production and accumulation of fibrotic tissue; 7) Production and release of increased levels of various cytokines and growth factors causing profibrotic and inflammatory effects; and 8) Epigenetic alterations including numerous changes mediated by non-coding RNAs. However, despite extensive investigation of the numerous pathogenetic events in SSc, it has not been established which of these processes is of primary importance or how they are temporally related during the development and progression of the disease.

## Pathogenesis of Systemic Sclerosis (SSc): overview

The current hypothesis of SSc pathogenesis proposes that the disease is initiated by unknown etiologic factors that may include toxic or chemical exposures, viral infections, microbial pathogens, or other still not identified mechanisms (10, 11). More specifically, the resemblance of SSc to fibrotic syndromes associated to toxic exposure such as polyvinyl chloride (12), contaminated rapeseed oil (Spanish toxic oil syndrome) (13), L-tryptophane contained products (eosinophilia myalgia syndrome) (14), and gadolinium (nephrogenic systemic fibrosis) (15), favors the implication of an environmental trigger, whereas the presence of an early type I interferon signature and activation of TLR8 by EBV genes found in monocytes from patients with SSc, suggest a possible viral etiology (16). In both scenarios, the activation of macrophages, monocytes and T-Cells are proposed to be the main mediators for the abnormal activation of the immune system (17). Another theory, states that under proper conditions and acting on a genetically receptive host, the causative initial mechanism(s) induces severe microvascular injury with profound structural and functional endothelial cell abnormalities and cause the development of frequently progressive cutaneous and internal organ tissue fibrosis. Although it is well recognized that SSc is not a genetic or genetically transmitted disease, the role of genetic factors is of crucial importance, as discussed extensively in numerous recent publications (18–21).

**Figure 1:**
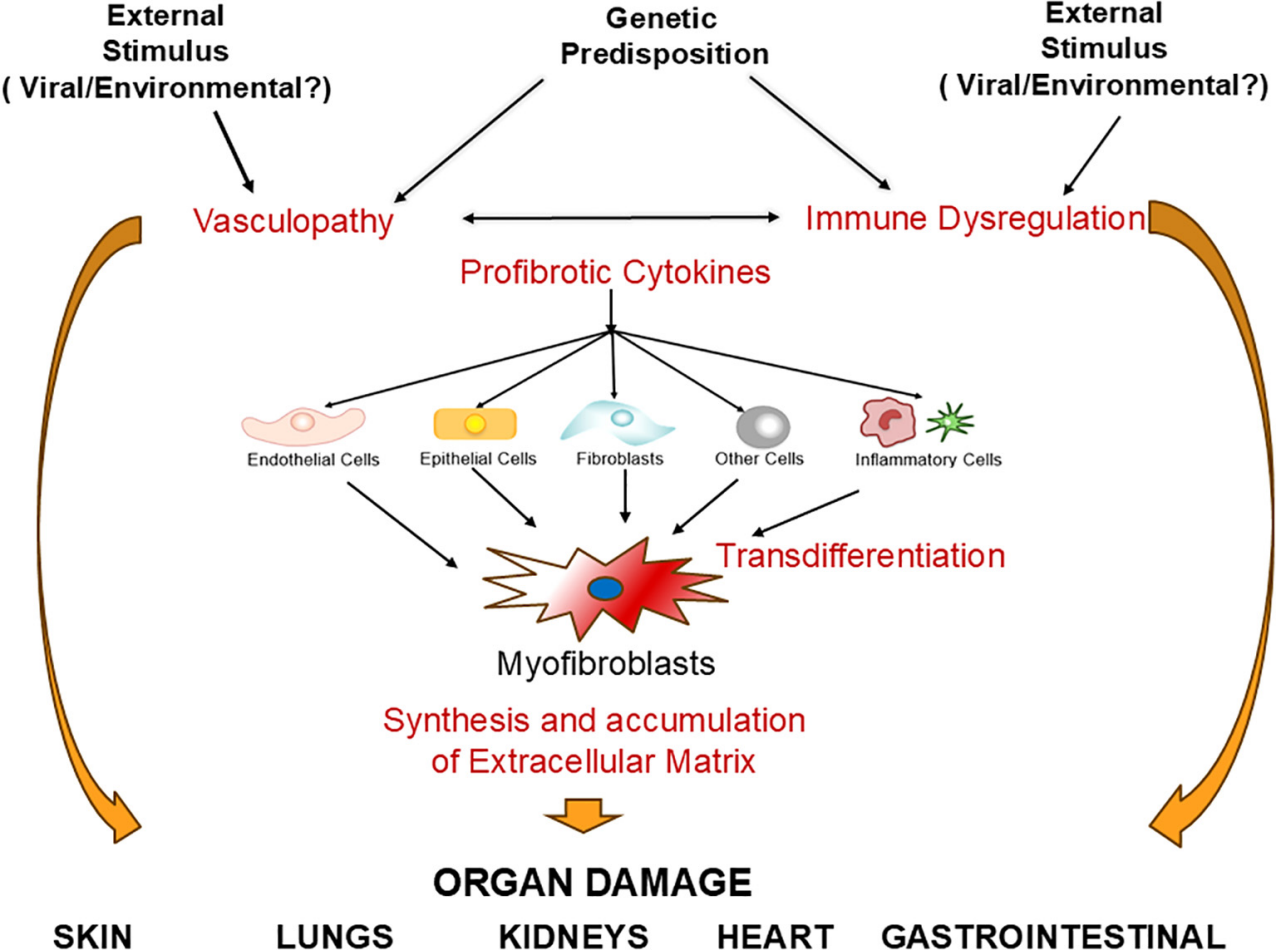
**Systemic sclerosis pathogenesis**. An unknown etiologic event (such as a virus or chemical substance) in a genetically predisposed host causes activation of multiple cell types including endothelial cells, inflammatory cells and fibroblasts. This activation process triggers abnormalities of the vascular tone causing vasospastic alterations, increases chemoattraction and adherence of monocytes/macrophages, promoting tissue inflammation and abnormal oxidative stress. An additional effect is the conversion of endothelial cells into myofibroblasts through Endothelial Mesenchymal Transdifferentiation (EndoMT) and the activation of other cells, including resident fibroblasts, circulating fibrocytes, epithelial cells (EMT), and adipocytes (AMT) into myofibroblasts. Increased myofibroblast numbers and metabolic activation cause increased production and accumulation of extracellular matrix molecules (ECM), which, coupled with microvasculopathy in multiple organs, and chronic inflammation are responsible for the most prominent SSc clinical manifestations in multiple target organs.

The prominent vascular involvement in SSc was initially described in early reports of SSc pathologic alterations that demonstrated the presence of intimal sclerosis and vessel wall hyperplasia in the arterioles of the kidneys and other affected visceral organs (22–24). Numerous subsequent studies confirmed the occurrence of vascular alterations as the earliest clinical manifestations of the disease, leading to the hypothesis that SSc was a vascular disease (25–27). The vascular alterations are clinically manifested as Raynaud’s Phenomenon, small artery vasculopathy, digital ulcers, multiple cutaneous and mucosal telangiectasias, and the development of severe fibroproliferative vasculopathy in multiple internal organs causing alterations in their function and their eventual failure (28–31). A second component of SSc pathogenesis is the recruitment of specific cellular elements resulting in the establishment of a chronic inflammatory process in the affected tissues. The participating inflammatory cells include dendritic cells, macrophages, T- and B- lymphocytes, and mast cells (32–36). The inflammatory cells initiate the production and release of numerous cytokines, chemokines, growth factors, and other molecular mediators that allow the establishment of the fibrotic process through multiple cellular interactions and cell-to-cell communications (37–41). The molecular effects induced by the cytokines and growth factors also cause remarkable cellular transdifferentiation events, including the phenotypic conversion of quiescent fibroblasts, epithelial cells, endothelial cells, and other cells such as pericytes and adipocytes into activated myofibroblasts, the cells ultimately responsible for the establishment and progression of the fibrotic process (42–48). This sequence of events (illustrated in **Figure 1**) results in the development of a severe and often progressive fibroproliferative vasculopathy, exaggerated and widespread accumulation of fibrotic tissue in the skin and numerous internal organs, and the establishment of a chronic inflammatory process in affected organs.

Here, we will review recent developments in the understanding of SSc pathogenesis and the involvement of microRNA (miRNA) alterations in SSc pathogenesis. Studies describing the role of inflammatory, humoral and cellular immunological abnormalities in SSc pathogenesis will not be discussed in detail here owing to the large number of publications and extensive reviews about these topics (18–21, 37, 44–50). We wish to emphasize, however, that the numerous and extensive publications related to SSc pathogenesis, preclude us from including in this review all the relevant studies on this important subject and we truly wish to express our deepest apologies to the investigators whose work was not specifically cited or discussed.

## Vascular and endothelial cell abnormalities in SSc

Vascular dysfunction is one of the earliest and most prominent clinical manifestations of SSc, as indicated by the occurrence of Raynaud’s Phenomenon and nailfold capillary microvascular alterations often preceding the appearance of other signs or symptoms of the disease. These early alterations are followed by the development of a systemic vasculopathy that results in abnormally dilated capillaries, microhemorrhages, vascular rarefaction and capillary loss, tissue injury caused by ischemia and hypoxia, cutaneous and mucosal telangiectasias, and fibroproliferative/occlusive vascular lesions in multiple organs (26–31). The visceral vasculopathy causes a spectrum of severe and even fatal clinical manifestations including scleroderma renal crisis (SRC), pulmonary arterial hypertension (PAH), interstitial lung disease, gastric antral vascular ectasia (GAVE), gastrointestinal dysmotility, myocardial dysfunction, erectile failure, and central retinal artery occlusion (51–59).

Following the vascular hypothesis to explain SSc pathogenesis proposed by Norton and Nardo (24) and by Campbell and LeRoy (25), this concept has been subsequently endorsed by numerous investigators (26–29), and it has become generally accepted that regardless of the putative SSc etiologic agent or event, microvascular alterations and endothelial cell injury and activation are central to the pathogenesis and the development of most of SSc clinical manifestations (60–62).

The initial events responsible for the vascular and endothelial cell injury in SSc are not fully known, although numerous putative factors have been suggested (Reviewed in Refs 62,63). Among these, the following have been most commonly considered: chemical and toxic agents, vasculotropic viral pathogens (10, 11), endothelial cell dysfunction (62), anti-endothelial cell antibodies (63, 64), and oxidative stress caused by reactive oxygen species (ROS) generated during episodes of ischemia/reperfusion (65). These factors either alone or in combination result in the development of crucial vascular and endothelial cell abnormalities that include: 1) Activation of endothelial cells; 2) Endothelial cell transdifferentiation into activated myofibroblasts, the cells responsible for the production of elevated amounts of collagens and other fibrotic macromolecules (48); 3) Production of increased levels of the potent profibrotic and vasoconstrictor polypeptide, endothelin-1, that besides its vascular effects, is a potent inducer of proliferation and ECM production by fibroblastic cells that also causes an exaggerated vasoconstrictor response resulting in further vascular hypoxia and endothelial injury (66–68); and 4) A complex imbalance of pro- and anti-angiogenic factors resulting in marked impairment of angiogenesis (69–71). The activated endothelial cells may also directly stimulate the profibrotic activities of various target cells such as vascular smooth muscle cells and fibroblasts and participate in the recruitment of other cells that may induce fibrotic alterations in the affected tissues. Collectively, the extensive endothelial cell and vascular alterations induce subsequent pathological events that maintain the vicious cycle of vascular injury, inflammatory response and tissue fibrosis that are the crucial components of SSc pathogenesis (5–9).

## Role of oxidative stress

Thirty years ago, Murrell proposed the hypothesis that elevated reactive oxygen species (ROS) caused abnormally increased systemic oxidative stress, which subsequently triggered cellular and molecular alterations that were responsible for the development of tissue fibrosis in SSc (65). Numerous subsequent studies have pursued this hypothesis and have provided strong support to the crucial role of oxidative stress in SSc pathogenesis. Indeed, it has been shown that elevated oxidative stress caused by ROS and reactive nitrogen species can stimulate the production of pro-fibrotic cytokines and growth factors (such as PDGF and TGF-β), induce proliferation and activation of fibroblasts, increase the expression and synthesis of type I collagen, and promote inflammatory changes and vascular dysfunction (72–77). Furthermore, it has been demonstrated that fibroblasts from affected SSc skin contain higher ROS levels and display markedly elevated activity of NADPH oxidases (NOX), particularly of NOX2 and NOX4 compared to fibroblasts cultured from skin from normal controls (78–81). It has also been shown that hypoxia induces alterations in the expression of several genes that participate in the phenotypic conversion of endothelial cells into activated myofibroblasts through a process of endothelial to mesenchymal transition (82).

Several *in vitro* studies have shown that reversal or reduction of the increased oxidative stress employing specific antioxidants or a NOX4 small molecule inhibitor abrogated these effects (78, 83). Furthermore, *in vitro* studies found a marked reduction in collagen expression levels in SSc dermal fibroblasts and in lung tissues from patients with SSc-associated ILD following treatment with antioxidant compounds (84, 85). These observations have been supported by several *in vivo* studies demonstrating that a decrease of ROS-generation caused suppression of fibroblast activation and abrogation of experimentally induced skin fibrosis (85). Several studies examined the effects of the anti-oxidant generator N-acetyl cysteine in SSc patients. One of these studies described a retrospective analysis of pulmonary function tests in SSc patients with pulmonary fibrosis following 24 months of intravenous N-acetyl cysteine. The treatment resulted in significant improvement in lung function, and the beneficial effects were greater in patients with early SSc-lung involvement (86). Another study was a randomized placebo-controlled trial on 25 patients with diffuse SSc without lung involvement (87). In contrast to the previous study (86), the results of this trial did not show any beneficial effects after 24 months of N-acetyl cysteine therapy (87).

Extensive experimental evidence has confirmed the occurrence of increased oxidative stress in SSc including the demonstration of elevated serum and plasma levels of various oxidative stress metabolic products such as 8-isoprostane, F2-iosprostane, malondialdehyde (MDA), and asymmetric dimethylarginine (ADMA); as well as elevated concentrations of DNA oxidation markers in the urine of SSc patients (Reviewed in Ref. 83). Furthermore, elevated levels of F2-isoprostane have been shown to correlate with the extent and severity of the SSc fibrotic process, particularly with lung fibrosis (88). A detailed mechanistic study assessed the molecular markers of oxidative stress in the serum of 36 diffuse SSc patients in comparison with 26 healthy controls performing quantitative measurement of reactive oxidative metabolites, including MDA and ADMA, total antioxidant capacity, lipid peroxidation, and evaluation of DNA oxidative damage (89). The results confirmed that total oxidative capacity and oxidative stress index were significantly increased in SSc patients as compared to healthy control subjects and demonstrated a correlation between these oxidative stress abnormalities and the presence of SSc pulmonary and gastrointestinal involvement. A remarkable observation in this study was that the oxidative stress abnormalities were also associated with the presence of anti-topoisomerase antibodies. Thus, this study confirmed the presence of molecular alterations indicative of excessive oxidative stress in the serum of SSc patients. However, it should be emphasized that the results did not show evidence of extensive lipid or DNA molecular oxidative damage and, therefore, it was suggested that SSc patients may have increased antioxidant capacity, although this possibility should require confirmation and validation in further studies (89).

Despite the published evidence of a strong association between fibrosis and oxidative stress/ROS, the pathways responsible are highly complex and have not been clearly delineated although there has been extensive investigation to elucidate the mechanisms involved. Among these studies, it was recently demonstrated that a novel oxidative stress pathway results in the induction of premature fibroblast senescence causing a marked increase in the production of inflammatory cascade mediators (90). Another recently identified mechanism was the demonstration that oxidative stress caused stabilization of specific kinase-phosphatase complexes that mediate activation of the SSc fibrotic process (91).

## Myofibroblasts: the effector cells in SSc tissue fibrosis

The progressive fibrotic process affecting the skin and numerous internal organs is one of the most distinctive clinical and pathological features of SSc. It has been generally accepted that this crucial pathologic alteration results from the accumulation in skin and other affected tissues of activated myofibroblasts. Myofibroblasts are mesenchymal cells first described in granulation tissue and were considered to be modified fibroblasts (92, 93). These cells display a markedly fibrogenic phenotype that is characterized by a persistent and exaggerated increase in the expression of genes encoding various interstitial collagens and other ECM proteins, the downregulation of genes for matrix-degrading enzymes, and the initiation of expression of contractile proteins such as α-SMA (43, 94). Myofibroblasts are considered the crucial cellular elements responsible for developing tissue fibrosis in SSc (95). The accumulation of activated myofibroblasts in affected tissues and the uncontrolled persistence of their elevated biosynthetic functions are considered to be crucial determinants of the extent, severity, and rate of progression of the fibrotic process in SSc (96–100).

### Cellular origins of myofibroblasts

There are multiple cellular sources responsible for originating the activated myofibroblasts present in affected SSc tissues. These include: 1) Proliferation and activation of tissue resident fibroblasts or perivascular and vascular adventitial fibroblasts and the selection of ECM over-producer and apoptosis resistant cells in response to specific signals from infiltrating inflammatory cells (101–103); 2) Recruitment of bone marrow fibrocytes, a unique class of fibroblast precursor cells expressing the CD34 hematopoietic/stem cell surface marker and able to produce type I procollagen and other ECM proteins typically expressed by fibroblastic cells (104, 105); 3) Transdifferentiation of endothelial cells to myofibroblasts, a process known as endothelial to mesenchymal transition (EndoMT) in which endothelial cells lose their phenotypic characteristics and acquire a myofibroblast phenotype and play a crucial role in the vasculopathy and fibrotic process in SSc (106–111); 4) Epithelial to mesenchymal transition (EMT), is another cellular phenotypic transition process (112–114) that has been postulated to contribute to the accumulation of myofibroblasts in SSc affected tissues (115–117); and 5) An additional source of myofibroblasts in affected SSc tissues are adipocytes and adipocyte precursor cells that may undergo a reverse differentiation process and acquire fibroblastic or myofibroblastic phenotype (118, 119). This process has been named “adipocyte myofibroblast transition” (AMT). The most important sources of activated myofibroblasts in SSc will be briefly described in the following sections.

### Increased proliferation of tissue fibroblasts

An increased number of cells capable of expression and production of ECM and other profibrotic macromolecules is a very important mechanism involved in the development and extension of the SSc fibrotic process. The crucial molecular pathways responsible cause an increase in the rate of proliferation of these cells and an increase in their viability and survival (95–97). Soluble mediators, either transported through the circulation or released by activated inflammatory cells present within the affected tissue infiltrates, induce a marked increase in the number of cells capable of producing excessive amounts of the molecular components of the fibrotic tissue. These effects may result from a direct action of the soluble mediators on the target cells or may be mediated by an autocrine stimulation of the production of profibrotic mediators from other cells. However, there is also an important, although less extensively studied, contribution of a reduction of cellular apoptosis and other cell death pathways, resulting in the prolongation of the cellular lifespan and in increased cellular survival (120–123).

### Migration of circulating fibrocytes

Fibrocytes are another important source of myofibroblasts. Fibrocytes are fibroblast precursor cells that play an important role in the development of tissue fibrosis (104, 105). These cells migrate from the bone marrow in response to specific chemokines released from the infiltrating inflammatory cells present in the affected tissues. These circulating bone marrow fibroblast precursor cells represent a unique cell population that is characterized by the expression of hematopoietic/stem cell surface markers, such as CD34 protein, coupled with the ability to synthesize type I procollagen and other ECM molecules typically produced and secreted by fibroblastic cells (103, 105).

### Cellular transdifferentiation

Cellular transdifferentiation is a highly complex biological process that results in the phenotypic conversion of fully differentiated somatic cell types into cells of another lineage. The molecular alterations involved in this highly complex transdifferentiation process have not been fully elucidated, although extensive investigation has shown that it is mediated by distinct mechanisms. These include changes in the expression and activity of specific protein transcription factors, involvement of complex protein interaction cascades, and the regulatory effects of various species of RNAs, including microRNAs (miRNAs) and long non-coding RNAs (lncRNAs). The main cellular transdifferentiation processes that may play a role in SSc-associated tissue fibrosis have been recently reviewed (48) and include: 1) Epithelial-Mesenchymal Transition, 2) Endothelial-Mesenchymal Transition, and 3) Adipocyte-Myofibroblast Transition. These processes will be briefly reviewed in the following sections.

### Epithelial-mesenchymal transition (EMT)

Epithelial-mesenchymal transition (EMT) is a complex cellular trans-differentiation process in which stationary and fully differentiated epithelial cells undergo profound changes in their cellular phenotype. These changes are characterized by the acquisition of mesenchymal cell features including the loss of cell-cell adhesion and apical-basal polarity, profound modifications in gene expression levels, marked morphological and cell shape alterations, and development of migratory capacity (112–114). Although EMT was extensively studied as an important mechanistic pathway involved in wound healing and cancer development and progression, extensive experimental evidence has demonstrated that this phenotypic cellular conversion participates in a large number of pathological processes, including a prominent role in the pathogenesis of SSc (115, 116), and of other autoimmune diseases (123).

The relevance of EMT to the pathogenesis of the SSc fibrotic process has been extensively demonstrated (115–117). Additional evidence includes the presence of increased nuclear translocation of myocardin-related transcription factor-A (MRTF-A), a factor responsive to mechanical stimuli that are key for EMT transformation in SSc patients (124). Furthermore, the secreted frizzled receptor protein 4 (SFRP4), a protein recently associated with EMT development, has been shown to be increased in the epidermis of SSc patients, and its serum concentration appeared to correlate with the degree of skin and lung fibrosis. These observations have suggested that SFRP4 should be considered as a biomarker of skin and lung fibrosis in SSc (123).

### Endothelial-mesenchymal transition (EndoMT)

EndoMT is a complex biological process in which endothelial cells lose their specific phenotype and progressively evolve into cells expressing mesenchymal characteristics acquiring a fusiform and elongated cell shape, cellular motility capabilities, and contractile properties (106, 107). At the molecular level, EndoMT results in the loss of endothelial cell-specific proteins including von Willebrand factor (vWF), CD31/platelet-endothelial cell adhesion molecule-1 (CD31/PECAM-1), and vascular-endothelial cadherin (VE-cadherin). There is a concomitant initiation of expression and production of mesenchymal cell-specific proteins including α-smooth muscle actin (α-SMA), extra domain A (EDA) fibronectin, N-cadherin, vimentin, fibroblast-specific protein-1 (FSP-1; also known as S100A4 protein), fibroblast activating protein (FAP), and fibrillar collagens type I and type III (106, 107). There has been extensive recent interest in the role of EndoMT in the pathogenesis of various human malignant, vascular, inflammatory and fibrotic disorders, and it has been demonstrated that it may play a very important role in the pathogenesis and in the development of the SSc-associated fibrotic process (108, 109) as well as in the vascular manifestations of numerous diseases (Reviewed in Ref. 110). Although most studies on EndoMT are related to the phenotypic conversion of arterial, venous, or capillary endothelial cells, recent studies have shown that lymphatic endothelial cells may also be able to trans-differentiate into myofibroblasts (125).

Extensive studies have shown that TGF-β1 and other TGF-β related growth factors, are the main inducers of EndoMT (126–129) and that these effects may be mediated by the nuclear translocation of the transcription factor Snail1 (127). Other studies have identified additional molecules that participate in the initiation, development and progression of fibrotic processes. One of these molecules is SOX9, a multifunctional transcription factor that plays a crucial role in chondrogenesis and skeletal tissue development and differentiation (130). SOX9 has also been shown to participate in numerous other regulatory functions in various tissues and organs and to play a role in various fibrotic diseases including idiopathic and SSc-associated pulmonary fibrosis (131). The mechanisms mediating the pro-fibrotic SOX9 effects have been recently examined. One extensive analysis provided evidence that SOX9 expression in human endothelial cells induced these cells to undergo EndoMT (132). It was further shown that these effects were mediated by alterations in the chromatin structure of the cells allowing histone modifications at previously silent binding sites causing a phenotypic change of the endothelial cells resulting in the initiation of expression of mesenchymal genes (132). These results allow to suggest the hypothesis that the initiation of EndoMT mediated by SOX9 expression in endothelial cells may play a crucial role in the development of various SOX9-mediated pathological fibrotic processes including pulmonary fibrosis, and SSc.

It should be emphasized, however, that given the great complexity of EndoMT and of the multiple and often redundant molecular mechanisms involved in its regulation, the overall effects of SOX9 may be quite variable, and highly dependant on the tissues affected as well as on the various pathophysiological conditions responsible for initiation and development of this process.

A highly relevant mechanism for the induction of EndoMT is tissue hypoxia (82, 133, 134). Although the molecular alterations involved have not been studied in detail, it has been shown that endothelial cells cultured under hypoxic conditions loose the expression of molecules that regulate the endothelial cell phenotype and acquire typical mesenchymal cell morphology and initiate the production of interstitial fibroblast macromolecules.

### Adipocyte and lipofibroblast to myofibroblast transition

Adipocyte-mesenchymal transition (AMT) is another cellular phenotype change that causes the conversion of mature adipocytes into pro-fibrotic myofibroblasts (135, 136). This process can be induced by exposure of adipocytes to TGF-β *in vitro*, resulting in the loss of the adipose specific markers (including PPAR-γ, perilipin, and FABP4), and promoting expression of fibrotic and mesenchymal proteins characteristic of the myofibroblast phenotype. Of remarkable importance to SSc pathogenesis were the observations that serum from SSc patients was able to induce the conversion of adipose tissue-derived stem cells into profibrotic myofibroblasts (118).

An additional cellular component related to ATM involves lipofibroblasts. Lipofibroblasts are fibroblast-like cells characterized by the content of abundant lipid storage deposits and the expression of adipose differentiation-related protein markers. These cells are abundant in the lungs and they also may undergo transdifferentiation into myofibroblasts, contributing to the activated profibrotic myofibroblast population during the initiation and development of SSc-associated lung fibrosis (136).

### Role of pericytes

Pericytes have an important role in vessel stabilization during angiogenesis (137), and have shown an increased expression of RGS5, an inhibitor of vessel maturation (138), and PDGFRβ along with decrease expression of αSMA. These activated immature pericytes are more abundant in the dermis of SSc patients (139) and can explain the defective angiogenesis seen in SSc despite of the presence of pro-angiogenic factors such as VEGF. In addition, it has been postulated that pericytes expressing PDGFβ+, NG2+, ADAM12+ can be precursors of myofibroblasts in skin from SSc patients (140).

## Role of inflammatory cells in tissue fibrosis in SSc

Besides tissue fibrosis, the presence of chronic inflammatory cells in affected SSc skin is one of the earliest and most consistent pathological alteration of the disease (32–34, 47). Numerous studies have shown that these cells, especially monocytes and macrophages, are capable of production and release of numerous profibrotic macromolecules including various cytokines and growth factors. These profibrotic cellular products induce quiescent fibroblasts in affected tissues to become active myofibroblasts and initiate their production of exaggerated amounts of interstitial collagens and other ECM macromolecules that result in pathologic and excessive fibrotic tissue accumulation (49, 50, 141, 142). Indeed, a detailed study employing coculture experiments of B cells and fibroblasts demonstrated that B cells were capable of direct stimulation of collagen and ECM protein synthesis by fibroblasts *in vitro* (143). This study also showed that B cell stimulation was further enhanced by BAFF (B cell activating factor), and it was suggested that these effects may be mediated by two distinct mechanisms, one resulting from cell-cell contact and the other induced by the B cell production of profibrotic molecules including IL-6 and TGF-β (143). Of substantial relevance to SSc pathogenesis are recent studies demonstrating that the products released by inflammatory cells also participate in the fibroproliferative vasculopathy characteristic of the disease (144).

Other studies have demonstrated that T cells secrete numerous cytokines, including IL-4, IL-6, IL-13, IL-17, and IL-31, that play an important role in SSc pathogenesis, including the stimulation of exaggerated synthesis and deposition of collagens and other fibrotic proteins by fibroblasts (46–50). IL-13 is an important product of CD8+ T cells, and its serum levels in SSc-affected patients were found to correlate with the presence and extent of skin fibrosis. Furthermore, it has been shown that IL-13 was able to stimulate collagen production in cultured SSc fibroblasts *in vitro*, and, therefore, it has been suggested to play a pathogenetic role in SSc-associated tissue fibrosis (145, 146). IL-31, is another cytokine that is also elevated in the serum of SSc patients. Recent studies have shown that IL-31 also stimulates collagen synthesis in dermal fibroblasts isolated from patients with SSc, and it has been strongly implicated in the development of SSc-associated Pulmonary Fibrosis (147–149). Indeed, it has been described that there is a highly significant negative correlation between serum IL-31 levels and the reduction of DLCO values in SSc patients (149).

## Role of growth factors in SSc tissue fibrosis

Numerous polypeptide growth factors play a crucial role in developing and extending the fibrotic process in SSc. The experimental evidence supporting the role of these growth factors in the pathogenesis of tissue fibrotic responses and the molecular mechanisms involved is very large and has been reviewed extensively in numerous publications (150–153). Therefore, we will only briefly discuss a few relevant recent studies related to the pathogenetic role of growth factors in the fibrotic process of SSc.

### Transforming growth factor beta (TGF-β)

It has become well recognized that TGF-β plays a crucial role in the initiation and progression of a large number of fibrotic processes (153, 154), and extensive studies have shown that it is of great relevance to the fibrotic process associated with SSc (151–153). These effects are mediated by multiple mechanisms including a potent stimulation of expression of genes encoding various interstitial collagens and other ECM macromolecules by fibroblasts and other mesenchymal cells (155–157), and the induction of cellular transdifferentiation of various types of cells into cells displaying very high expression of various collagens (Reviewed in Ref. 48).

The production and activation of TGF-β are highly regulated and involve numerous complex pathways. TGF-β is initially produced in a latent form in which the polypeptide is bound to a latency-associated peptide (LAP) and is subsequently secreted to the extracellular compartment as a large complex with a carrier protein called latent TGF-β binding protein (LTBP). Once released from the LTBP, the TGF-β molecules become a bioactive dimeric complex that is able to bind to different isoforms of TGF-β receptors (TβR) located in the cell membrane of TGF-β-responsive cells and initiate intracellular signaling pathways that are context dependent and transduce the signal from the cell surface to the nucleus resulting in a potent modulation of expression of multiple genes (158–164). The intracellular molecular cascades initiated following TGF-β binding and receptor activation have been extensively described in multiple publications (155–161) and will not be discussed here in further detail.

Extensive research studies have shown that the main signal transducer of TGF-β fibrotic signaling is a family of proteins known as Smad proteins that play an essential role in the regulation of the TGF-β-induced fibrotic response (164–166). Other regulatory pathways of TGF-β effects involve various non-receptor tyrosine kinases (167, 168), including the cytoplasmic Abelson kinase (c-Abl) and protein kinase C-δ (PKC-δ), to contribute to the fibrosis and vasculopathy of the skin and internal organs in SSc. These effects are most likely mediated by the crucial participation of c-Abl in TGF-β-induced EndoMT, an effect mediated by the cooperative interaction with PKC-δ (169, 170).

### Connective tissue growth factor (CTGF)

CTGF, also known as CCN2, is a cysteine-rich protein with pleotropic effects that has recently been recognized as an important mediator of normal and pathological tissue fibrotic responses (171–173). Numerous studies have demonstrated the potent profibrotic effects of CTGF (172), and it has been considered to play a crucial role in the SSc fibrotic process and to correlate with the extent and severity of tissue fibrosis (174–177). Some of these effects are of substantial relevance to SSc pathogenesis owing to the fact that vascular wall smooth muscle cells are the main targets of these effects (178), and therefore, it is possible that CTGF may contribute to the development of Raynaud’s Phenomenon and other vascular alterations characteristic of SSc.

### Platelet-derived growth factor (PDGF)

The PDGF family of growth factors plays an important role in the development and maintenance of normal connective tissue, and abnormalities of their signaling pathways may be involved in the pathogenesis of multiple diseases (Reviewed in Ref. 183). Numerous studies have described the involvement of PDGF and related molecules in the pathogenesis of fibrotic diseases, including SSc (179–182). Elevated expression of PDGF and its receptors has been found in SSc skin and lung tissues and there is evidence that TGF-β stimulates the expression of the PDGF receptor, PDGFRα, in SSc cells suggesting that cross-talk between TGF-β and PDGF pathways may regulate tissue fibrosis in SSc (180) and it has been suggested that PDGF/PDGF receptor may represent a novel molecular target for SSc (182). Indeed, pharmacologic inhibition of these pathways has been shown to halt the SSc associated fibrotic process (182).

### Fibroblast growth factors (FGFs)

The FGFs comprise a family of twenty two polypeptide growth factors grouped in seven subfamilies collectively characterized by their ability to induce potent mitogenic effects that play important roles in development, angiogenesis and wound healing (183, 184). Numerous studies have demonstrated the mitogenic effects of FGF during inflammatory and fibrotic responses often potentiating the pro-fibrotic effects of TGF-β, although some recent studies have described that some of the members of the FGF family may cause antifibrotic effects that may be mediated by inhibition of TGF-β pathways (185, 186). Regarding the role of FGF in SSc pathogenesis, it has been shown that basic FGF is increased in the skin of SSc patients (187). However, the precise role of FGFs in the initiation of progression of the fibrotic process in SSc has not been completely elucidated and further studies will be required to conclusively determine the contribution of these potent growth factors to the pathogenesis of fibrosis in SSc.

### Vascular endothelial growth factor (VEGF)

VEGF is an endothelial cell specific growth factor mediating key signals for angiogenesis including stimulation of endothelial cell proliferation and differentiation, and modulation of endothelial permeability (188–190). Quantitative analysis of serum levels of VEGF in patients with SSc and healthy controls showed that serum VEGF levels were significantly higher in SSc patients and correlated with the extent and severity of skin fibrosis and nailfold capillary loss, suggesting that high VEGF levels may promote in the capillary damage in SSc and may correlate with the extent and severity of the fibrotic process and with disturbed angiogenesis (191–193).

### Insulin-like growth factors (IGFs)

Although early studies identified IGFs as the main stimulators of sulfate incorporation into cartilage (194, 195), several recent studies have shown that IGFs display strong profibrotic effects. Studies related to their role in the SSc fibrotic process have shown elevated levels of serum IGF-I and IGFBP-3 in SSc patients that correlated with the extent of skin involvement and the presence of pulmonary fibrosis (196). Furthermore, IGF-I mRNA was found to be upregulated in the affected skin tissues of patients with SSc. Subsequent extensive studies demonstrated that IGF-II is involved in SS-associated pulmonary fibrosis and is a potent inducer of collagen production and other fibrotic pathways and that these effects are mediated by multiple mechanisms, including an increase in the expression of pro-fibrotic signaling molecules, a decrease in the expression of several collagen degradation enzymes, and an increase in the differentiation of fibroblasts into myofibroblasts (197, 198).

## Role of miRNAs in the regulation of SSc-associated fibrosis

MicroRNAs (miRNAs) are small (~22 nucleotides), evolutionarily conserved non-coding RNA, which play important roles in the regulation of the expression of a large number of protein-coding genes at the post-transcriptional level (199). The mechanisms involved in post-transcriptional miRNA regulation of gene expression are complex and require the sequence-specific complementary binding to the 3’ untranslated region (UTR) of target mRNAs, suppressing their expression by either inhibiting mRNA translation or facilitating mRNA degradation of their corresponding mRNA (199, 200). Non-coding RNAs can also induce potent cellular transdifferentiation effects (201).

Several non-coding RNAs have been shown to be involved in SSc tissue fibrosis, displaying either profibrotic or antifibrotic effects (202–204). Among the most extensively studied miRNAs in the pathogenesis of SSc are miR-21 and miR-29 (201–203) and it has been shown that elevated expression of miR-21 is associated with stimulation of fibroblast proliferation and increased production and accumulation of various ECM proteins. Furthermore, it has been demonstrated that miR- 21 expression is upregulated by TGF-β1 and may represent one of the profibrotic molecular effects of the growth factor on dermal fibroblasts. The miR-29 family comprises several distinct miRNAs that play a crucial role in the regulation of the fibrotic process and are capable of potent anti-fibrotic effects including regulatory modulation of several fibrosis-related genes such as the genes encoding collagens type I, II and IV, fibronectin, and laminin, as well as various enzymes involved in tissue remodeling, including TIMP and other matrix metalloproteinases. Indeed, it has been shown that miR-29 inhibits the TGF-β1/Smad signaling pathway and suppresses the TGF- β1-induced pro-fibrotic process. The expression of several members of the miR-29 family is reduced in SSc and in other tissue fibrosis diseases and the extent of their reduction has been shown to inversely correlate with the extent and severity of the fibrotic process (205, 206).

Recent studies have found the scaffold long non-coding RNA (lncRNA), HOTAIR, was found to be overexpressed in SSc in dermal fibroblasts, inducing a histone EZH2-dependent increase in collagen production and the expression of the myofibroblast marker α-SMA *in vitro*. In addition, histone-mediated repression of miRNA-34A expression was also observed, with the subsequent activation of the NOTCH pathway (207).

Another lncRNA that has been associated with the strong IFN-I signature in SSc is the X-inactive specific transcript (XIST), which is involved in X chromosome inactivation (XCI) in female mammals. Plasmocytic dendritic cells (pDC) in SSc overexpress TLR8, contributing to keeping a strong IFN-I signature in skin and lung tissues. Escaping from silencing by incomplete XCI in inflammatory cells from women may affect TLR7/8 signaling. The decreased expression of XIST and of the transcriptional repressor SPEN in SSc pDCs, suggests that an altered XCI at the TLR7/8 locus may, indeed, contribute to IFN-I chronic activation mediated by pDCs (208). In addition to that effect, TSIX, the XIST antisense, can also play a more direct role in SSc fibroblasts, activating endogenous TGF-β signaling and may play a role in stabilizing collagen RNA and upregulating collagen in these cells (209).

Upregulation and downregulation of multiple non-coding RNAs have been described in SSc (205–234), and it is very likely that although the evidence is not conclusive, these RNAs may be shown to be highly relevant to the SSc fibrotic process. A list of the non-coding RNA associated with SSc fibrosis is shown in **Table 1**. Multiple other miRNAs have been implicated in SSc vasculopathy and immune dysregulation. Although the scope of this review is focused on fibrosis, this subject has been recently reviewed (202, 235).

**Table 1:** MicroRNA implicated in SSc fibrosis.

miRNA	Target	Levels in SSc	References
21	SMAD7	Overexpressed	(219)
26a-5p	Collagen	Underexpressed	(220)
27a-3p	SPP1 and ERK	Underexpressed	(221)
29a	COL3A1	Underexpressed	(222)
30b	PDGFR-β	Underexpressed	(223)
31	EndoMT, TGF-β	Overexpressed	(224)
33a-3p	DDK-1	Overexpressed	(225)
92a	MMP-1	Overexpressed	(226)
129-5p	Connective Tissue Growth Factor	Underexpressed	(227)
142-3p	Integrin αV	Overexpressed	(228)
150	Integrin β3	Underexpressed	(229)
155	EndoMT Wnt,	Overexpressed	(230)
196a	Collagen	Underexpressed	(231)
202-3p	MMP1	Overexpressed	(232)
483-5p	Collagen	Overexpressed	(214)
4458	?	Overexpressed	(233)
Let-7a	Collagen	Underexpressed	(234)

## Concluding remarks

SSc is a highly complex autoimmune disease of unknown etiology, causing severe clinical and pathological alterations in affected individuals that often manifest as progressive cutaneous and multiple organ fibrosis and associated generalized obstructive vasculopathy. Despite the severity of the disease, there is no curative therapy at the present time, however, it is expected that further knowledge about its pathogenesis may provide effective therapeutic approaches. This review outlines the current understanding of the disease pathogenesis and may be of value to the development of effective treatment for this serious autoimmune pathologic condition.
